# Nanomedicine for Neurodegenerative Disorders: Focus on Alzheimer’s and Parkinson’s Diseases

**DOI:** 10.3390/ijms22169082

**Published:** 2021-08-23

**Authors:** Keelan Jagaran, Moganavelli Singh

**Affiliations:** Nano-Gene and Drug Delivery Group, Discipline of Biochemistry, University of KwaZulu-Natal, Private Bag X54001, Durban 4000, South Africa; 215055447@stu.ukzn.ac.za

**Keywords:** neurodegenerative disorders, gene therapy, nanomedicine, Parkinson’s disease, Alzheimer’s disease

## Abstract

Neurodegenerative disorders involve the slow and gradual degeneration of axons and neurons in the central nervous system (CNS), resulting in abnormalities in cellular function and eventual cellular demise. Patients with these disorders succumb to the high medical costs and the disruption of their normal lives. Current therapeutics employed for treating these diseases are deemed palliative. Hence, a treatment strategy that targets the disease’s cause, not just the symptoms exhibited, is desired. The synergistic use of nanomedicine and gene therapy to effectively target the causative mutated gene/s in the CNS disease progression could provide the much-needed impetus in this battle against these diseases. This review focuses on Parkinson’s and Alzheimer’s diseases, the gene/s and proteins responsible for the damage and death of neurons, and the importance of nanomedicine as a potential treatment strategy. Multiple genes were identified in this regard, each presenting with various mutations. Hence, genome-wide sequencing is essential for specific treatment in patients. While a cure is yet to be achieved, genomic studies form the basis for creating a highly efficacious nanotherapeutic that can eradicate these dreaded diseases. Thus, nanomedicine can lead the way in helping millions of people worldwide to eventually lead a better life.

## 1. Introduction

Neurodegenerative disorders, through the subsequent immune activation of the central nervous system (CNS), impose substantial burdens on the public and health sectors. While immune activation may aid in regeneration and repair, together with various other mechanisms such as the limitation of neurotropic viral infections and the removal of necrotic cells, it can also lead to the development of neurodegeneration, ischaemia, infections, and immune-mediated disorders. Neurodegeneration is defined as the slow but gradual degeneration of neurons and axons in the CNS that results in abnormalities in cellular function and, consequently, cellular demise [1]. The ensuing symptoms stem from the degeneration stage, beginning with a loss of coordination and memory, to a complete loss of the ability to function as a normal healthy individual. The three major neurodegenerative disorders have been identified as Alzheimer’s disease (AD), Parkinson’s disease (PD), and amyotrophic lateral sclerosis (ALS) [2]. These diseases are closely linked to environmental cues, disordered immunity, advancing age, and the genetic make-up of the affected individual [3].

Although over 50 million people are believed to be affected by AD globally, these numbers are progressively increasing due to the increased average lifespan and genetic and environmental factors. An estimated 152 million of the population are projected to be affected by the year 2050 [4]. This will create an immense global economic strain in the years to come. Beyond AD, and based on the Parkinson’s Foundation Prevalence Project, approximately 10 million individuals have presented with PD, with about one million belonging to the United States of America (USA) alone. These patients succumb to the high costs involved in their treatment over and above the complete alteration of their normal lives. This economic burden is estimated to be close to USD 52 billion per year in the USA [5]. The pathogenesis of the diseases above is initiated through the accretion, aggregation and alteration of the normal host proteins, the modification of tissue homeostasis, disrupted blood flow, and immunological damage [6,7]. To date, treatment of these diseases remains palliative, majorly as a result of the inability of therapeutic drugs and biomolecules to effectively traverse the blood–brain barrier (BBB) [8]. The limitations arise through the brain microvessel endothelial cells (BMVECs), creating brain capillaries. The high concentrations of drug-metabolizing enzymes and the expression of outward drug efflux transporters, together with low pinocytic potential and close intercellular junctions, restricts the passage of biomacromolecules and low molecular weight compounds from the blood to the brain [3]. The goal of optimal therapeutic outcomes stands firm in creating site-specific and safe therapeutic means of treating these life-changing diseases effectively.

This challenge of crossing the BBB could potentially be overcome via the utilization of novel therapeutic nano-delivery systems. Nanomedicine, the amalgamation of nanotechnology and medicine, involves the use of nanoscale-sized particles to safely and efficiently transport pharmacologically active agents (therapeutic drugs or genes) to diseased sites in the body, including the brain. Furthermore, exploiting the synergy between nanomedicine and gene therapy may hold greater promise in treating various diseases, including monogenic disorders. Genetic alterations can characterize the onset of AD and PD. Comprehensive knowledge of these genetic aberrations is imperative in creating a treatment strategy that is both effective and specific to the type of the disease without harming normal body cells. Several genetic disorders, including cancer therapy, are being investigated by nanomedicine-based interventions, the clinical success of which will be determined in time. This review paper explores the possibility of utilizing nanomedicine and its potential role as an effective therapeutic strategy against two selected neurodegenerative disorders, Alzheimer’s and Parkinson’s disease. 

## 2. Neurodegenerative Diseases

Neurodegenerative diseases form part of a heterogeneous group of disorders characterized primarily by the progressive and slow degeneration of the structure and function of the CNS. The classification of the various disease types is due to their clinical manifestations, such as cognitive and behavioural disorders, with pyramidal and extrapyramidal movement disorders being the most common [9]. There is gradual neuronal dysfunction and death due to oxidative stress, neuroinflammation, programmed cell death and proteotoxic stress, with abnormalities noted in the lysosomal/autophagosomal and ubiquitin–proteasomal systems [9].

**Figure 1:**
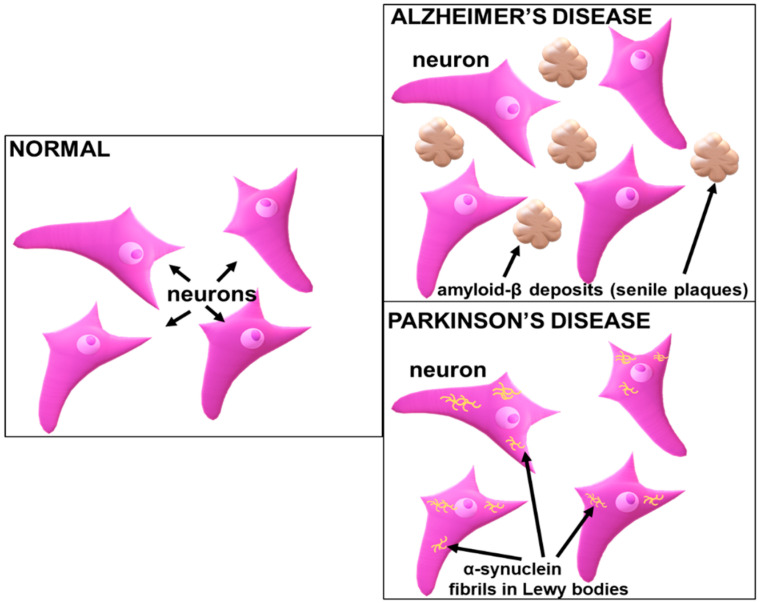
The appearance of amyloid plaques in Alzheimer’s disease and α-synuclein inclusions in the neocortical neurons in Parkinson’s disease.

While protein abnormalities define most neurodegenerative disorders, the clinical manifestations often only present themselves a while later, with more than one disease process being observed in a patient [10]. Furthermore, diagnostics are hindered due to a lack of available biomarkers, with the exception of rare cases where genetic mutations are identified as the causative agents [11]. The most common protein abnormality disorders are tauopathies, transactivation response DNA binding protein 43 (TDP-43) proteinopathies, amyloidosis, and α-synucleinopathies. The neuroanatomical and cellular distribution, together with abnormalities present in the protein conformations of the relevant disorders, constitute the histopathological features that allow for the neuropathological diagnosis of the disorder [9]. In AD, amyloid-β deposits (senile plaques) (Figure 1) are often seen in the neocortex, while tau inclusions such as a neurofibrillary tangle manifest in a neocortical neuron. In PD, α-synuclein inclusions (Figure 1) are commonly found in the neocortical neurons [12]. Unlike neuronal inclusions present in viral infections that contain foreign viral proteins, the aggregation of the abnormal proteins is composed of various cellular components and intrinsic neuronal proteins. These abnormalities have more amyloid-like properties, with filament formations and β-pleated sheet-enriched secondary structures [9]. The aggregation of proteins in these disparate disorders portray templating, nucleation, multiplication, growth and spread. A reduction in these amyloidogenic proteins can regulate the concentration of the targeted proteins, thereby effectively impeding the growth and nucleation [13]. Since amyloid-β and tau proteins are reportedly discharged into the extracellular space, which is regulated by the activity of the neurons, targeting the cause of the disease progression, they may abrogate this discharge by halting their cellular release, transport, and uptake into cells, or by arresting their movement between cells. Hence, novel nano-therapeutics can be formulated to overcome the limitations of the current therapy while incorporating gene therapy to restrict the disease progression. 

To date, there is no cure available for these neurodegenerative disorders, with current treatment remaining palliative. Western medicine has provided relief to the symptoms experienced by patients suffering from these disorders, ranging from dopaminergic treatment for movement disorders related to PD to antipsychotic medication prescribed for dementia/AD to treat the psychological and behavioural symptoms [14,15]. To date, there are several drugs available that treat the symptoms of AD and PD (Table 1 and Table 2). While some relief is noted in patients, the ever-progressing disease renders the drug therapy inadequate and ineffective [15]. Beyond this, the major limitation in drug therapy is the unfavourably low concentration of the drug that eventually localizes in the CNS following the systemic administration. This is primarily due to the blood–brain barrier (BBB), which hinders the effective transport of the drugs to the brain [16]. 

While there are many neurodegenerative disorders that affect individuals, AD and PD are two of the most highly prevalent disorders, accounting for a significant portion of the global burden in respect to CNS disorders. These diseases can occur through genetic predisposition or sporadically through the interaction of genes with the environment. Genes involved in neurodegeneration, the metabolism of xenobiotics and the function of dopaminergic neurons have been observed to be associated with PD. In contrast, a polymorphism on the susceptibility gene of the epsilon 4 allele, apolipoprotein E gene (APOE), is strongly related to the onset of AD [17]. The use of nanomedicine and gene therapy could pave the way for novel treatment strategies for CNS-related diseases. 

### 2.1. Parkinson’s Disease (PD) and Implicated Genes

In contrast to diseases such ALS, leukodystrophies, and lipid storage diseases, PD exhibits a confined, well-defined, and compact group of affected neurons. Studies pertaining to PD have been made simpler due to the readily available rodent and primate models. Knowledge of the survival, function, and development of dopamine neurons is also well understood [20]. PD is primarily caused by neuronal damage in the substantia nigra (SN) of the brainstem, diminution of the nigrostriatal neurotransmitter and dystrophy of the associated projection fibres to the corpus striatum, leading to motor system malfunction that progresses to non-motor symptoms [20,21,22]. These affected cells are responsible for synthesizing dopamine and the control of movement via the makeup of the innervate large area of the forebrain. Manifestations of symptoms are underappreciated prior to 60% loss of the substantia nigra pars compacta (SNpc) dopamine neurons, which causes a drastic loss (80%) of dopamine [23,24]. Early symptoms present as tremors, slowness of movement, rigidity, and walking difficulties, together with apathy, anxiety, and depression [25].

Genetic links in PD have been previously elucidated, with the first discovered genetic defect or mutation being present on the SNCA gene on chromosome 4, encoding for α-synuclein [26]. Further investigation revealed a duplication or triplication of SNCA that suggested that overexpression of α-synuclein could lead to toxicity and PD [27]. Other genes that have been identified include Leucine-Rich Repeat Kinase 2 (LRRK2), DJ-1, ubiquitin carboxyl-terminal esterase L1 (UCHL 1), phosphatase and tensin homolog (PTEN), and Parkin [21]. The most common monogenic mutation noted is in the LRRK2 gene, particularly mutations in Gly2019Ser, observed in patients presenting with autosomal-dominant PD. This mutation type accounts for 1% of sporadic and 4% of familial PD worldwide. Apart from mutations in the Gly2019Ser, other mutations were noted in Arg1441His, Arg1441Cys, Arg1441Gly, ile2020Thr, Tyr1699Cys and Asn1437His. This particular gene has various protein–protein interaction domains, together with an enzymatic core that brings about GTPase and serine–threonine kinase activities. The gene possesses the Gly2019Ser mutation in the kinase domain, adjacent to the Ras-of-complex (Roc) GTPase domain, which results in an unfavourable increase in the LRRK2 kinase activity [28]. It is due to this that current gene therapy techniques utilize this domain as a target for LRRK2 kinase inhibitors. 

DJ-1, encoded by the PARK7 gene, is a highly conserved protein made up of 189 amino acids that are expressed under physiological conditions, initially linked to early-onset PD of a familial nature [29]. It was earlier reported that a mutation in the DJ-1 protein resulted in a disease with parkinsonian clinical manifestations such as tremors, dyskinesia, and rigidity [30]. Hence, the PARK7 gene serves as an interesting target for gene therapy, since it functions to protect against oxidative stress and is under-expressed in PD. Studies related to increasing DJ-1 levels to obtain neuroprotection of the dopaminergic neurons have been verified using rat PD models [31]. Alternatively, recombinant fused TAT cells have also been used to cross the BBB and reduce 6-hydroxydopamine (6-OHDA) toxicity during intrastriatal administration [32].

Functions of the ubiquitin–proteasome system (UPS) are maintained by ubiquitin, and mutations therein are suggested to be involved in PD. This was observed in a study of Lewy bodies of the nerve cells in PD patients, identifying mutations in the UCHL1 gene as being the cause [33]. The decreased expression of this gene resulted in the degradation of axons, motor ataxia, and instability of the free ubiquitin levels. This gene, containing 223 amino acids and accounting for 1–2% of human brain protein, is majorly expressed in the peripheral nervous system [34]. In contrast, mutations in the UCH-L1S18Y gene portray specific antioxidant protective functions that potentially decrease the risk of developing PD. Hence, there is a strong potential for the application of gene therapy and/or nanomedicine in patients presenting with UCHL1-related PD [35,36].

Phosphatase and tensin homolog (PTEN) with tumour suppressor properties serves as a dual-specificity phosphatase with lipid and protein phosphatase activities. It is also a regulator of the PI3K/AKT pathway [37]. However, cells with the overexpression of PTEN portray an activation in the proteolytic cascade for apoptosis, which has been correlated to a decline in cell survival kinase AKT, leading to neuronal damage and death [38]. It is important to note that increased AKT can reduce oxidative stress levels and cell death, and therefore serves a therapeutic role in reducing neuronal damage. Furthermore, PTEN-induced kinase 1 (PINK1) has portrayed a significant role in oxidative DNA damage, preventing mitochondrial oxidative stress, autophagy, and mitochondrial conservation. It, therefore, reverses the apoptotic function of overexpressed PTEN in neuronal cell damage [39,40]. 

The PARK2 gene is noted as the most common autosomal recessive juvenile form of PD and is a significant contributor to sporadic and familial early-onset PD [41]. Parkin is encoded by the PARK2 gene and is characterized by a ring-between-ring domain E3 ubiquitin ligase, which is responsible for the catalysis of covalently attached ubiquitin to specific substrates and the regulation of vital cellular processes such as apoptosis and mitochondrial quality control [42,43,44]. A loss of Parkin function results in the ubiquitination of mitochondrial proteins downstream of the PINK1 kinase, resulting in mitophagy [45]. The build-up of these dysfunctional and damaged mitochondria causes oxidative stress, resulting in the loss of dopaminergic neurons and the occurrence of the motor symptoms of PD [41].

Hence, gene knockdown of overexpressed genes or the expression of under-expressed genes (in the case of Parkin) may be possible using nanoparticles to carry the gene of interest or therapeutic gene to target specific areas of the brain. While it may be complex, understanding the genetics pertaining to PD is highly imperative in designing an efficacious treatment at the genetic level. Since individuals present with varied genetics, ‘personalized medicine’ using therapeutic gene strategies holds great promise, especially for reducing side effects.

### 2.2. Alzheimer’s Disease (AD) and Implicated Genes

AD is one of the most common irreversible causes of dementia, a progressive neurodegenerative disease that manifests as a gradual loss of cognitive skills and memory. AD accounts for 60–80% of dementia cases [46]. The neurodegeneration occurs as a result of extracellular β-amyloid plaque deposition and intracellular neurofibrillary tangle deposition that cause neurotoxicity and synaptic loss [47]. The amyloid precursor protein (APP), presenilin 1 (PSEN1), and presenilin 2 (PSEN2) genes are predominant in the autosomal dominant forms of AD, while apolipoprotein E (ApoE) is evident in sporadic AD. The pathogenesis of AD exhibits various mechanisms, with APP processing into amyloid-beta peptide (Aβ) via the β-secretase and γ-secretase complexes being identified as the main mechanism and a potential target for therapeutics [48]. This could involve the use of inhibitors of APP-cleaving enzymes, such as caspases, meprin-β, rhomboid-like protein-4 (RHBDL4), membrane-type matrix metalloproteinases (MT-MMPs/η-secretases) and legumain (δ-secretase) [48,49].

Mutations in the PSEN1 gene are the most common cause of familial AD (FAD). This gene encodes for presenilin-1 (PS1), which is responsible for the catalysis of γ-secretase, which cleaves some type 1 transmembrane proteins, such as Notch and APP [50]. Before the cleavage mentioned above, cleavage occurs via the β-secretase to generate Aβ. This results in the production of Aβ40 and Aβ42, with the latter product being hydrophobic and nucleating Aβ aggregation in the brain, leading to amyloid plaque deposition. This has been reported as the pathogenetic mechanism causing FAD [51]. Since the natural properties of presenilin are involved in memory and learning, together with neuronal survival following age progression, it was suggested that a mutation in PSEN1 leads to the loss of presenilin function, resulting in dementia and neurodegeneration in FAD [50].

In contrast to PSEN1, PSEN2 mutations are rare, with less than 40 mutations identified. Besides the progression of AD with age, environmental aspects are proposed to play a major role in the autosomally inherited form of AD [51]. Mutations in this gene are responsible for early-onset AD (EOAD), late-onset AD (LOAD), frontotemporal dementia (FTD) and dementia with Lewy bodies (DLBs), together with other diseases such as dilated cardiomyopathy (DCM) and breast cancer [37,52]. The pathogenetic mechanism related to mutations in PSEN2 and PSEN1 are similar, but with Aβ42 aggregation occurring to a lesser extent for PSEN2 [53]. Both PSEN 1 and PSEN2 may be ideal candidates for therapeutic intervention via gene therapy and nanomedicine. 

ApoE is responsible for lipid transport and injury repair in the brain. Polymorphisms related to this gene predispose an individual to AD [54]. Although most mutations occur at the ε3 allele, the ε4 allelic mutations pose a greater risk to AD and present an increased risk for age-related cognitive decline and cerebral amyloid angiopathy [55]. ApoE permits the delivery of lipids and Aβ through the subsequent binding to cell-surface receptors on the brain. Hence, mutations result in the initiation of toxic events leading to neurodegeneration through synaptic dysfunction. Furthermore, ApoE is responsible for the regulation and clearance of Aβ aggregation, glucose metabolism, mitochondrial function, brain lipid transport, neuroinflammation and neuronal signalling, with mutations significantly affecting individuals [54].

## 3. Nanoparticles and Nanomedicine

**Figure 2:**
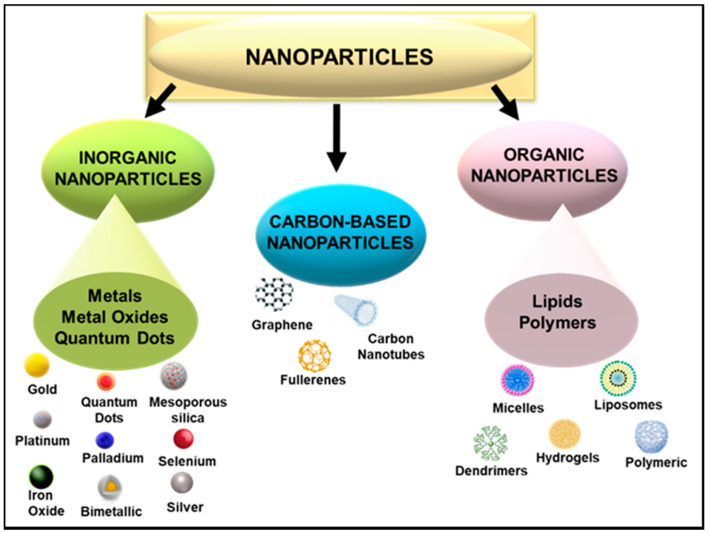
Broad classes of nanoparticles showing those commonly used in nanomedicine.

The use of nanoscale particles in medicine, especially as carriers of therapeutics, holds great potential for treating many diseases, owing to their many favourable properties such as size, shape, and surface morphology [56]. Nanotechnology further permits intentional design variations, providing the ability to control their properties [37]. This malleability of nanoparticles (NPs) allows for the attachment of various biomolecules, thereby allowing for the efficient and safe transportation of pharmacologically active agents, such as genes or drugs. NP delivery vehicles of 1–100 nm in size have the ability to penetrate significant physiological barriers such as those found in the lungs, liver, gastrointestinal fluid, blood, tumour vasculature, mucosal membranes, and the blood–brain barrier [57,58,59]. Various NPs have been utilized in this regard, each portraying their unique characteristics as a therapeutic, diagnostic or theranostic tool. The ability to conjugate therapeutic nucleic acids and drugs to NPs has opened up avenues in target-specific nanomedicine. Apart from medicine, NPs can be employed in cosmetics, packaging, electronics, and biotechnology. NPs can be broadly classed as organic, carbon-based, or inorganic NPs (Figure 2).

Biocompatibility relies on the physicochemical properties of the NPs, with each NP displaying distinctive properties. The modification of NPs with polymers and targeting ligands can enhance binding affinities with the gene or drug being conjugated [60], in addition to cell-specific uptake. The noble metals, gold (Au), silver (Ag), platinum (Pt), and palladium (Pd), have been commonly employed due to their favourable physiochemical, biological, and optical properties [58,61]. The physicochemical properties of AuNPs are easily tunable for clinical application [62]. They have demonstrated promising results in various diseases, including smallpox, cancer, syphilis, AIDS, and skin ulcers [63], and have also been used to detect copper ion-induced aggregated Aβ peptides [64]. AgNPs possess anti-microbial and anti-viral properties that have been exploited as a pre-treatment for wound infections [65]. The use of unmodified AgNPs as delivery vehicles has been hindered by their propensity to aggregate and increase in size [66]. Pd is more commonly used in dentistry, where it is part of the composition of electrical equipment [61,67]. Bimetallic Au-Pd NPs modified with quercetin have been studied as possible inducers of autophagy in Alzheimer’s disease [68]. Pt is a good antioxidant for the reduction in free radicals [58] and is part of the anticancer drugs cisplatin and oxaliplatin, which have reported some neurotoxicity [69].

Selenium (Se), an essential micro-element, is required by all organisms for various biological functions, with the supplementation of Se being reported to reduce the incidence of cardiovascular diseases, osteoarthritis, type 2 diabetes, and neurodegenerative diseases such as AD [70,71]. Se NPs possess favourable properties, including anticancer and antioxidant properties of Se, while exhibiting lower cytotoxicity, better bioavailability, biocompatibility, and biodegradability in vivo [71,72]. Due to their potential synergistic effect with the therapeutic gene or drug, these NPs are becoming increasingly popular. The application of mesoporous silica NPs (MSNs) as nano-delivery vehicles has gained significant momentum due to their porous structures that offer both inner and outer increased surface areas for therapeutic cargo [73,74]. This porous nature of MSNs allows for the possible combination delivery of therapeutic genes and drugs, which can improve biological activity [75]. Quercetin-encapsulated silica NPs have demonstrated potential against Cu--induced oxidative stress observed in neurodegenerative diseases [76]

Iron oxides, commonly referred to as magnetic NPs (MNPs), including maghemites, magnetites and ferrites, have been widely studied in nanomedicine due to their low cytotoxicity, biodegradability, stability, magnetization, biocompatibility, low sensitivity to oxidation and reactive surfaces, non-carcinogenicity, and ease of synthesis and modification [77]. The target-specific delivery of MNPs can be achieved by the process of magnetofection that uses an external magnetic field to guide their delivery. Their application has been extended to magnetic hyperthermia, magnetic resonance imaging (MRI), and delivery systems [78,79]. However, unmodified MNPs are hydrophobic, and can aggregate and generate reactive oxygen species, limiting their in vivo efficacy [80]. 

Quantum dots (QDs) have unique optical properties, but due to their composition, which often includes metals such as cadmium and zinc, they tend to be toxic. This could be overcome using modified core–shell QDs or coated QDs [75]. Carbon nanotubes, either single-walled or multi-walled, can readily enter cells. However, without either internal or external functionalization, they are insoluble, cytotoxic, hydrophobic, and immunogenic [81]. The use of polymeric delivery systems has evolved over the years, with cationic polymers being favoured due to their ability to bind anionic molecules such as nucleic acids. In addition, the chosen polymers must be biocompatible, biodegradable, and stable in vivo [75]. Hence, polymers such as dendrimers have been popular due to their many cationic groups. They have been further utilized as suitable stabilizers of metallic NPs such as AuNPs [82,83]. Poly (lactic-co-glycolic acid), a polymer approved by the Food and Drug Administration (FDA), has shown good properties for use in drug delivery in combination with Au [84], while its PEGylated derivatives have been investigated in AD [85]. From the lipid-based NPs, liposomes have been commonly used for the delivery of bio-active compounds, with some positive results noted in animal models for AD [86,87]. 

**Figure 3:**
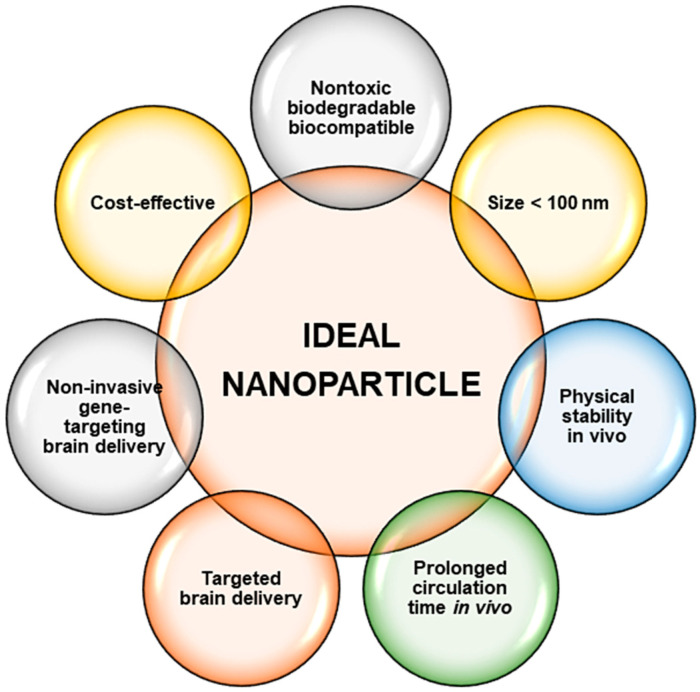
Ideal criteria required for the development of a safe and efficient nanoparticle for use in nanomedicine.

Overall, inorganic NPs in most cases possess an advantage over their organic counterparts, especially with regard to the ease of synthesis and functionalization approaches, size, stability, and their theranostic potential. All the NPs mentioned above have shown potential in nanomedicine and may be extended to neurological disorders such as AD and PD. Overall, for these nanosystems to be suitable, predetermined properties of the NP needs to be addressed and prioritized [88], as illustrated in Figure 3.

### 3.1. Challenges Facing Nanoparticles

The use of NPs does not come without challenges, especially when considering their use as therapeutic delivery vehicles for neurodegenerative diseases. Besides the BBB, which poses the greatest hindrance to therapeutics, neurotoxicity due to nano-delivery systems also raises safety concerns [89]. This neurotoxicity is commonly noted by the generation of oxidative stress, and predominantly depends upon the morphology, size, surface area, solubility, concentration, and the duration and mode of the nanotherapeutic administration [90]. Although some metals play pivotal roles in the human body, the accumulation and aggregation of metal NPs may be a cause for concern. Using the neuronal model of PC12 cells, it was earlier reported that iron NPs produced significant cytotoxicity [91], while manganese and Cu NPs generated reactive oxygen species [92]. The use of zinc oxide NPs induced apoptosis in neural stem cells [93], while the oral administration of Ag NPs was toxic and accumulated in the kidney, liver, and brain in Sprague Dawley rats [94]. In addition, the administration of iron oxide NPs to mice models produced oxidative stress, neurodegeneration [95], cell-cycle-dependent neuronal apoptosis [96], and neurobehavioural toxicity [97]. 

Despite these challenges, the physicochemical properties of NPs, as mentioned previously, make them attractive candidates in nanomedicine. To overcome some of these challenges, the NP formulations must encompass biocompatible materials that are also biodegradable and readily excreted from the system [98]. Their ability to cross the BBB is further described in Section 3.2. In addition, the toxicities evidenced are often dependant on the type of NP used, with surface functionalizations being a way forward in reducing adverse effects and interactions. Hence, there is no “one size fits all” regarding the choice of a NP and its application. It is essential to identify the advantages and disadvantages related to the use primarily of metals and non-metal carriers, bearing in mind that many metals are required in the body, as mentioned previously. Hence, the concentration utilized will be critical to maintaining homeostatic equilibrium. The use of targeted approaches in treating AD and PD will be crucial, as cell-specific targeting is essential for treating damaged or mutated genes while upholding the integrity of normal functioning genes and cells. However, it is clear that a deeper investigation of NPs is warranted when formulating therapeutics for the CNS. Presently, there is a dearth of information on NP neurotoxicity, suggesting an urgent need for further studies both in vitro and in vivo to provide a foundation around which future studies can be designed. Using emerging technologies, especially in silico studies, computer and mathematical modelling, together with greater knowledge in bioinformatics, may assist in the challenges facing nanomedicine in the formulation of an ideal NP. 

### 3.2. Crossing the Blood–Brain Barrier

**Figure 4:**
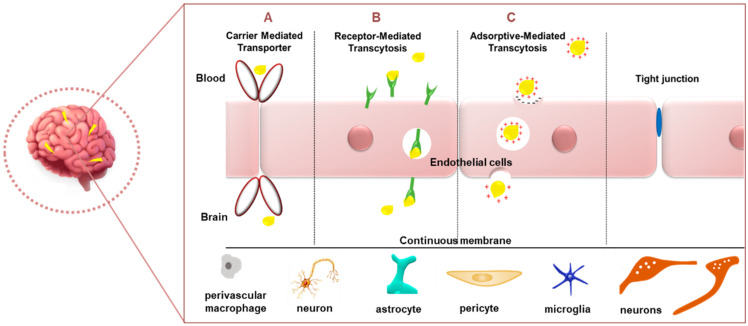
Common mechanisms for passage through the BBB. (**A**) Carrier-mediated transporter, (**B**) receptor-mediated transcytosis and (**C**) adsorptive-mediated transcytosis.

The blood–brain barrier (BBB) is a dynamic boundary that serves a self-protective role in modulating the transport of biomolecules from the blood into the brain while obstructing the ingress of toxic chemicals and larger drugs. While this role is greatly beneficial, it serves as an obstacle to current therapeutics. The BBB, a specialized part of the vascular system, is made up of a basal lamina comprising extracellular matrix proteins (laminin, heparan sulphate or collagen), together with endothelial cells, pericytes, astrocyte endfeet, and interneurons [99]. The vascular, neuronal, and glial cells are known to interact, forming a cellular network appropriately termed the neurovascular unit that is involved in the maintenance of tissue homeostasis [100]. The BBB is the largest barrier in the CNS and has a surface area of 20 m^2^. It is considered a critical site for the exchange of molecules between the blood and the CNS [101]. Since NPs are small (mostly <200 nm) molecules, they have the advantage of being able to traverse this BBB. Apart from size, properties such as charge, especially a positive charge, suitable surface functionalizations, the addition of targeting ligands such as cell-penetrating peptides and polyethylene glycol for improved circulation time in vivo imbue NPs with the capacity to successfully cross the BBB [99]. It has been observed that molecules penetrate the brain via the carrier-mediated transporter (CMT) (Figure 4), which includes the glucose transporter (GLUT1), adenosine transporters (CNT2), large neutral amino-acid transporters (LAT1), and monocarboxylic acid (MCT1) [8]. Drug delivery of chemo-nanotherapeutics in the treatment of brain diseases portrayed the use of circulating cells, such as exosomes, erythrocytes, neutrophils, and leukocytes, which possess the ability to spontaneously cross the BBB [102]. 

Other means of entry can be seen in receptor-mediated transcytosis (RMT) and adsorptive-mediated transcytosis (AMT) (Figure 4) [103]. The former transport system relies on the NPs’ ability to be modified to possess ligands permitting the efficient binding to receptors present at the BBB. Ligands can be directed to targets such as GLUT1 or albumin transporters [104], lactoferrin (Lf) receptors, LRP1 (using angiopep-2) [105], or transferrin receptors (TfR) (using transferrin ligand) [106]. TfR has been identified to be sometimes overexpressed in neuronal [107] and glioma cells [108]. However, the levels of brain transferrin decrease with age and a dramatic reduction is observed in neurodegenerative diseases such as AD or PD [109]. However, the TfR does offer great promise in the delivery of therapeutic agents across the blood–brain barrier to the brain [110]. RMT thereby exploits the role of surface-labelled nanocarriers for the efficient entry of the nanocomplexes into the brain. However, the choice and concentration of the attached ligand will be limiting factors that will determine the success of endocytosis. Gold nanospheres [111] and gold nanostars conjugated to a cell-penetrating peptide demonstrated the ability to cross the BBB [112].

AMT, however, portrays a slightly varied mechanism of action in that it utilizes electrostatic interactions between the negatively charged BBB and the positively charged NPs [91]. It was reported that gold-NP-decorated wheat germ agglutinin was taken up by nerve terminals and retrogradely transported by the axons to the CNS [113]. These carrier transporters all allow for the slackening of the BBB surfactants, thereby disrupting the endothelial cell junctions and allowing for the entry of the NPs into the brain. Studies in mice models have reported a lack of damage to the brain [114,115]. However, it has been noted that the choice of the in vivo disease model for testing the NPs’ ability to cross the BBB is crucial, since BBB permeability may differ from rodents to humans [99]. Extensive studies on transport molecules enable researchers to create therapeutics that can exploit the natural physiological barriers for the safe and efficient delivery of pharmacologically active agents to the brain. The optimal parameters for a nanocomposite to be able to pass through the BBB were proposed to be a low molecular weight (<400 Da), a suitable charge, log *p* < 2, non-ionization, the presence of hydrogen bonds (8–10), and lipophilicity [100].

Besides the use of NPs in drug delivery, which has shown some in vivo instability and immune reactions due to the intravenous administration of the nanosystem, the employment of gene therapy utilizing NP carriers can be considered.

### 3.3. Gene Therapy

The idea of gene therapy dates back to the 1960s and is the treatment or prevention of a disease or a genetic disorder using therapeutic nucleic acids [116]. Despite the high transfection rates obtained using viral delivery vehicles, the disadvantages relating to a low loading capacity, large-scale manufacturing, the size of gene it can carry, and the safety factors of potential oncogenicity and immunogenicity prompted the development of non-viral methods. Non-viral gene delivery systems have a greater ability to target cells/tissues, a significantly lowered oncogenic and immunogenic nature, an enhanced efficacy of preparation at low cost, no limitation on the size of the genetic cargo, and amenability to structural manipulations [117]. From the non-viral delivery vehicles, cationic polymers and lipid-based constructs, especially cationic liposomes, have been the most studied to date, with the use of inorganic NPs now gaining momentum. 

NPs can overcome both intracellular and extracellular barriers that hinder gene delivery. These barriers include nuclear uptake, the avoidance of clearance by the reticuloendothelial system (RES), endosomal and lysosomal escape, the protection of genetic cargo from degradation, nucleic acid release, and the targeting of specific cells [118]. Due to inorganic NPs portraying greater surface area to volume ratios with tunable magnetic, optical, and biological properties, they can be engineered to deliver genes with enhanced efficacy by modifying the shape, chemical composition, and size. An ideal gene delivery vehicle should possess properties such as the ability to disrupt the endosomal membrane, to cross the plasma membrane, to bind, condense and protect the nucleic acid cargo, to ensure target specific delivery, have stability in circulation, and be able to evade the immune system [118,119]. 

The extensive research about the pathogenetic mechanisms of neurodegenerative disorders has led to the identification of specific genetic defects implicated in the progression of diseases. Gene therapy permits the delivery of genomic cargo, which includes microRNA (miRNA), small interfering RNA (siRNA), guide RNA (gRNA), and messenger RNA (mRNA). Studies portrayed success in gene silencing strategies via RNA interference (RNAi), which utilizes siRNA, miRNA and piwi-interacting RNA to decrease the synthesis of the targeted mRNA molecules [120]. When synthetic double-stranded siRNAs (21–25 nucleotides in size) are transfected into mammalian cells, they target the specific mRNA sequences with a high degree of specificity, which leads to gene silencing [75]. The RNAi revolution has opened up a novel avenue for therapeutic intervention in a wide array of disorders, from cancer to neurodegenerative diseases [75,121]. Overall, the successful application of siRNA-mediated gene silencing in medicine would require a suitable delivery vehicle, preferably a nanocarrier, that would ensure the safe and efficient delivery of the siRNA. Genome editing has been recently introduced into gene therapy, and heralds a technique that can directly target aberrant genetic changes at diseased sites [122].

A potential target in gene therapy is the abnormal accumulation of misfolded proteins such as amyloid β-oligomers and α-synuclein (Figure 1), which generate endoplasmic reticulum (ER)-associated degradation and ER stress [123]. The aggregation of these proteins in the ER lumen consequentially causes a destabilization of the ER calcium homeostasis and distortion in the unfolded protein response (UPR) signalling, resulting in neuron death via pro-apoptotic responses [124,125]. This can be overcome by targeting the UPR signalling to enhance protein folding, as seen when PD was treated by targeting the reduction in dopaminergic neuron apoptosis and improving the motor performance, thereby delaying the disease progression. This was permitted via gene therapy, which involved targeting the overexpression of the BiP (glucose-regulated protein 78) gene, which is linked to a reduction in the unfolded protein response [126]. Hence, gene silencing strategies can be successful in such cases.

Furthermore, mitochondrial respiratory dysfunction has been noted in diseases such Huntington disease (HD), AD, PD, and ALS, resulting in the limited regulation of mitochondrial quality, NAD+ depletion, oxidative damage, protein aggregations, disrupted ATP synthesis and unbalanced mitochondrial calcium homeostasis [127,128,129]. Gene therapy has been seen to overcome this phenomenon via either inhibiting the mitochondrial damage or promoting mitochondrial biogenesis. Alternatively, neurotoxicity in experimental HD and PD can be regulated by the overexpression of regulators of mitochondrial oxidative stress and dynamics, including PGC-1α, HSP70, TFEB [130,131].

Other mechanisms of pathogenesis are seen in abnormal rapamycin (mTOR) signalling in PD, AD and HD, together with epigenetic dysregulation, autophagy, and microglial and astrocyte dysfunction [132]. Each mechanism exhibits unique modes of dysfunction owing to the progression of the disease, and it is therefore important to understand which mechanism is involved in a patient presenting with these diseases to administer the appropriate treatment with maximum efficacy. Furthermore, gene therapy has proved its efficacy in various other diseases. It, therefore, is a great contender for neurodegenerative therapeutics, following research on genetic aberrations in patients with PD and AD.

Table 3 highlights a few of the NPs used for gene therapy of the CNS from 2017 to 2020. The success of such experiments has expanded the knowledge of nanomedicine in neurodegenerative disorders, aiding in the specific targeting of the causative genes or aggregated proteins. Gene therapy strategies delivered using nanoparticle vectors are attractive alternatives as they can potentially satisfy many requirements for safe and efficient delivery across biological barriers, especially the blood–brain barrier. Aside from the advantages portrayed in gene therapy, the biological synthesis of NPs vaunts its own array of benefits with regard to specific extracts utilized [133] that may work synergistically with the therapeutic gene.

### 3.4. Nanomedicine in Clinical Trials—Update

Several clinical trials using drugs as secretase inhibitors and therapeutic antibodies in AD have been conducted, with only a few being completed, and the majority discontinued [8]. Interestingly, there has been a global lack of novel drug development for AD since 2003 [138]. This was also evident in a recent search of the NIH library, with only two studies related to NP delivery. One entitled “Safety, tolerability and efficacy assessment of intranasal nanoparticles of APH-1105, a novel alpha-secretase modulator for mild to moderate cognitive impairment due to Alzheimer’s disease” is only due to start in 2023. The second trial, “A Phase 2, pilot open-label, sequential group, investigator blinded study of magnetic resonance spectroscopy (31P-MRS) to assess the effects of CNM-Au8 for the bioenergetic improvement of impaired neuronal redox state in Parkinson’s disease”, began in December 2019, and was scheduled to be completed in July 2021 [139]. This study utilized gold nanocrystals. Although gold nanocrystals have been approved recently for treating multiple sclerosis [140], updates on the current study are awaited. Positive results can only propel the use of NPs in future investigations.

## 4. Conclusions

Nanomedicine is emerging as a highly efficacious tool to overcome barriers that still challenge traditional medicine. The combination of nanomedicine and gene therapy can be exploited for greater therapeutic benefits. This review highlighted some of the genes involved in the disease progression of PD and AD that may open the prospect of gene therapy studies. A greater understanding of the causes of genetic aberrations and how they lead to neurodegeneration can lead to tailored therapeutics in response to a specific mutation type presented by an individual. While a cure may not be immediate, such research studies form the stepping stones to ultimately create a treatment strategy that would one day eradicate diseases linked to neuronal damage and help millions of patients worldwide to live a normal and healthy life. The combination of nanomedicine and neuroscience can potentially provide novel solutions to many CNS-related disorders, including AD and PD. The array of nanoparticles currently available needs to undergo stringent testing as to toxicity and stability and must be optimized for gene or drug delivery to the CNS.

## Figures and Tables

**Table 1:** Common drugs employed to treat the symptoms of Alzheimer’s disease (adapted from [18]).

Drug	Function
Donepezil (Aricept)	Cholinesterase Inhibitor
Galantamine (Razadyne)	Cholinesterase inhibitor
Tacrine (Cognex)	Cholinesterase inhibitor
Memantine (Namenda)	N-methyl-d-aspartate receptor blocker
Rivastigmine (Exelon)	Cholinesterase inhibitor
Memantine extended-release and donepezil (Namzaric)	N-methyl-d-aspartate receptor and acetylcholinesterase inhibitor

**Table 2:** Common drugs used to treat the symptoms of Parkinson’s disease (adapted from [19]).

Drug	Function
Carbidopa-levodopa (Sinemet, Parcopa, Rytary, Duopa)	Dopamine decarboxylase inhibitor/DA precursor
Levodopa (Inbrija)	Dopamine precursor
Entacapone (Comtan)	Cathechol-o-methyltransferase inhibitor inhibits the breakdown of Levodopa
Tolcapone (Tasmar)	Cathechol-o-methyltransferase inhibitor inhibits the breakdown of Levodopa
Opicapone (Ongentys)	Cathechol-o-methyltransferase inhibitor inhibits the breakdown of Levodopa
Carbidopa/Levodopa Entacapone (Stalevo)	Dopamine decarboxylase inhibitor/DA precursor/COMT inhibitor
Pramipexole (Mirapex)	Dopamine agonist
Ropinirole (Requip)	Dopamine agonist
Apomorphine (Apokyn, Kynmobi)	Dopamine agonist
Rotigotine (Neupro)	Dopamine agonist
Selegiline (Eldepryl, Zelapar)	Monoamino oxidase-B inhibitor; inhibits breakdown of dopamine
Rasagiline (Azilect)	Monoamino oxidase B inhibitor; inhibits breakdown of dopamine
Safinamide (Xadago)	Monoamino oxidase-B inhibitor; inhibits breakdown of dopamine
Amantadine (Symmetrel, Gocovri, Osmolex)	Mixed mechanisms, including N-methyl-D-aspartate antagonism
Istradefylline (Nourianz)	Adenosine 2A antagonist
Trihexyphenidyl (Artane)	Anticholinergic
Benztropine (Cogentin)	Anticholinergic

**Table 3:** Application of nanoparticles in neurodegenerative studies since 2017.

Nanoparticle	Disease	Therapeutic	Therapeutic Effect	Cell Entry	Ref
Gold	PD	*pDNA* incorporated exogenous interfering RNA (RNAi) and Nerve growth factor (NGF)	Inhibition of PC12 cells and substantia nigra striatum dopaminergic neuronal apoptosis	Nerve growth factor (NGF) endocytosis	[134]
Gold	AD	3.3 nm L- and D-glutathione	Inhibition of Aβ42 aggregation	Chiral nanoparticle endocytosis across the BBB	[133]
Silver	PD	Citrate cap	Up-regulation of hydrogen sulphide (H_2_S) and Ag_2_S—reducing neurotoxicity	Natural properties of silver penetrate the brain	[135]
Silver	AD	*Lampranthus coccineus* and *Malephora lutea* F. Aizoaceae plant extract	Anticholinesterase and antioxidant activity as a plant-based anti-Alzheimer drug	Natural properties of silver penetrate the brain	[136]
Selenium	AD	Curcumin-loaded nanospheres	Decrease in amyloid-β plaques in AD lesions	Curcumin’s capability to bind with amyloid b and iron in plaques by intermolecular hydrogen bonds without any additional chemical linkers	[137]

## Data Availability

Not applicable.
